# Supplementary material for: Single-cell multi-omics analysis reveals IFN-driven alterations in T lymphocytes and natural killer cells in systemic lupus erythematosus
Source: Wellcome Open Res. 2022 May 12;6:149. Originally published 2021 Jun 14. [Version 2] doi: 10.12688/wellcomeopenres.16883.2 (PMC9046903; doi:10.12688/wellcomeopenres.16883.2)
Supplement: Peer review history

# Peer review: Reviewer response for version 2

**DOI:** 10.21956/wellcomeopenres.19813.r50490

**Published:** 2022-05-16

**Authors:** Camilla Jandus, Mara Cenerenti

**Affiliations:** 1 Department of Pathology and Immunology, Faculty of Medicine, University of Geneva, Geneva, Switzerland

I very much appreciate the efforts of the authors to address my concerns.

Is the work clearly and accurately presented and does it cite the current literature?

Partly

If applicable, is the statistical analysis and its interpretation appropriate?

Yes

Are all the source data underlying the results available to ensure full reproducibility?

Yes

Is the study design appropriate and is the work technically sound?

Partly

Are the conclusions drawn adequately supported by the results?

Yes

Are sufficient details of methods and analysis provided to allow replication by others?

Yes

Reviewer Expertise:

CD4 T cells, cytotoxicity, cancer, Innate lymphoid cells

We confirm that we have read this submission and believe that we have an appropriate level of expertise to confirm that it is of an acceptable scientific standard.

# Peer review: Reviewer response for version 1

**DOI:** 10.21956/wellcomeopenres.18624.r49546

**Published:** 2022-04-27

**Authors:** Parth Narendran

**Affiliations:** 1 Institute of Immunology and Immunotherapy, University of Birmingham, Birmingham, UK

This is a nice piece of work using RNA and protein profiling of T and NK cells in 2 SLA patients with high and low interferon signatures and a non-SLA control patient. 

 The authors accepted that the patient numbers are low, and that this is essentially a proof-of-concept study. There is certainly merit in increasing the patient numbers to validate this work, but this does not detract from the proof-of-concept findings of the current analysis.

 My main comment relates to the disease state of the IFNhi patient and the implications for biomarker discovery. Does the IFN signature relate to current SLA disease activity or to future prognosis? The context here is that there are established clinical scoring criteria for scoring disease activity in SLE (e.g., Thanou *et al*., 2019 ^1^ ), therefore if the findings of this study (ideally replicated in a larger cohort) apply to current disease state, then the question arises as to whether it adds anything more to what is obtained through simple clinical scoring. These are questions that can be explored through larger cohort analysis. If however the multi-omics analysis brings additional information about prognosis, this would be a more intriguing outcome of this analysis. 

 Minor comments relate to the length of the discussion which can be more focused.

Is the work clearly and accurately presented and does it cite the current literature?

Yes

If applicable, is the statistical analysis and its interpretation appropriate?

Yes

Are all the source data underlying the results available to ensure full reproducibility?

Yes

Is the study design appropriate and is the work technically sound?

Yes

Are the conclusions drawn adequately supported by the results?

Yes

Are sufficient details of methods and analysis provided to allow replication by others?

Yes

Reviewer Expertise:

Clinical endocrine disorders and immunology of type 1 diabetes

I confirm that I have read this submission and believe that I have an appropriate level of expertise to confirm that it is of an acceptable scientific standard, however I have significant reservations, as outlined above.

## References

[1] : Scoring systemic lupus erythematosus (SLE) disease activity with simple, rapid outcome measures. *Lupus Sci Med* .2019;6(1) : 10.1136/lupus-2019-000365 e000365 10.1136/lupus-2019-000365 31921432PMC6937422

# Response

**Published:** 2022-05-04

**Authors:** Ricardo Ferreira

**Affiliations:** University of Oxford, UK

We appreciate the Reviewer’s feedback and suggestions. We detail below the replies to the specific concerns raised by the reviewer:

  

- 1/ My main comment relates to the disease state of the IFNhi patient and the implications for biomarker discovery. Does the IFN signature relate to current SLA disease activity or to future prognosis? The context here is that there are established clinical scoring criteria for scoring disease activity in SLE (e.g., Thanou *et al*., 2019 ^1^ ), therefore if the findings of this study (ideally replicated in a larger cohort) apply to current disease state, then the question arises as to whether it adds anything more to what is obtained through simple clinical scoring. These are questions that can be explored through larger cohort analysis. If however the multi-omics analysis brings additional information about prognosis, this would be a more intriguing outcome of this analysis.

*Disease activity scores in SLE have been widely implemented and reflect the high degree of clinical heterogeneity of the disease. The manuscript that the reviewer highlights (Thanou et al 2019) provides a very good summary of the diversity of different disease activity scores in SLE and, reassuringly, demonstrates good concordance between them. The need to harmonise the disease activity score between the patients is particularly important for treatment purposes, but often overlooks the heterogeneity of clinical manifestations between patients with active disease.*

*Because of this challenge, the search for clinical biomarkers in SLE has been a topic of intense research. The best available biomarker in SLE is currently the detection of an IFN signature in blood, which has been shown to be correlated with disease activity scores - albeit not very strongly. In the past we have investigated how different biomarkers such as soluble SIGLEC-1, soluble CD25 or IL-2 concentrations were correlated with both disease activity scores and the classical IFN signature, and have shown that patients with high IFN signature scores are associated with increased disease activity and renal disease involvement (see Oliveira J et al, Arthritis Res Ther. 2018; doi: 10.1186/s13075-018-1649-1). However, it is clear that there is vast clinical heterogeneity, and that no marker is currently sufficient to properly stratify SLE patients.*

*The main aim of applying single-cell multi-omics technologies in SLE is exactly to better understand the different biological mechanisms that underpin the disease and whether we can better stratify patients from treatment. In this proof-of-principle study, we wanted to investigate whether a more quantitative targeted single-cell multi-omics approach would reveal distinct alterations in the cellular composition of the T and NK cell compartment in SLE patients. For this we deliberately opted to select two extreme patients in terms of the IFN signature score that we had previously quantified. Of note, as we remark in the manuscript, both SLE patients profiled in this study were recruited outside of* *their regular clinic visits and were therefore assumed to have good disease management at the time of sampling. This strongly suggests that the cellular alterations that we report in this study, as well as the IFN signature in the IFNhi patient profiled in this study may not be associated with active disease activity. This is not unexpected and underscores the relatively low correlation between the transcriptional IFN signature and disease activity score.*

*Our proof-of-concept study in a very small cohort of patients suggests that our targeted single-cell multi-omics approach can detect substantial alterations in the T and NK cell subsets of SLE patients, and therefore supports the application of this approach in a much larger (and clinically characterised) cohort of SLE patients. As the reviewer rightly observes (and as stated above in the replies to reviewers #1 and #2), the ideal experimental setting would be to apply this approach on a prospective longitudinal cohort with ancillary disease activity information. This experimental design would allow to investigate whether the cellular alterations identified by the single-cell multi-omics analysis are indeed prognostic or reflective of flares of disease activity. Our study shows that such analysis is warranted and may provide better insight into the different pathogenic mechanisms that underlie SLE.*

  

- 2/ Minor comments relate to the length of the discussion which can be more focused.

*We agree that the discussion is long and requires focussing. We have edited the discussion to reduce the size and improve the focus.*

# Peer review: Reviewer response for version 1

**DOI:** 10.21956/wellcomeopenres.18624.r49283

**Published:** 2022-04-01

**Authors:** Camilla Jandus, Mara Cenerenti

**Affiliations:** 1 Department of Pathology and Immunology, Faculty of Medicine, University of Geneva, Geneva, Switzerland

This study describes the profiling of immune cell subpopulations in SLE, using a combined transcriptomic and proteomic methodology, down to the single-cell level.

 The main weakness of the study is the very limited number of subjects, which dampens the enthusiasm of the reviewer. Conclusions are drawn on 1 donor per study population (1 patient IFNhi, 1 patient IFNlo, 1 HD, with only limited clinical characteristics provided). It would have been highly recommended to validate the findings obtained by the transcriptomic/Proteomic analyses in additional samples from IFNhi and IFNlo SLE patients, by a flow cytometry based methodology.

 Moreover, while the authors repeatedly stress the fact that they are utilizing a novel method combining protein and mRNA quantification, this type of analysis has also been largely used by others, eg., CITE-seq, on unsorted single immune cells, with good resolution and identification of discrete, rare immune cell states (such as CD4 CTLs).

 Finally, while the inclusion of a "stimulated" condition appears very interesting to the reviewer, the rationale to use PMA/Ionomycin instead of aCD3/CD28 activation (at least for T cell subsets) is unclear. This would have provided a more physiological activation, that would better reflect the in vivo events occurring in patients.

**Minor comments:**

- By considering NK cells as CD56+CD127+ CD3- cells, the authors are including helper ILC3s in their sorting. This might represent a confounding parameter for the results' interpretation.
  Similarly, CD25-CD127+ CD4 T cells will also include Naive CD4 T cells, while in Fig 1B these cells are named Teff.
- Though nicely represented, the readability of several Figure panels is difficult (numbering of clusters Fig 2A, gene names and scale bar Fig 2B, blurry volcano plot Fig 3B,...).
- The discussion is very long, and highly speculative based on the findings obtained in the small patients cohort. I suggest refinement, specifically the sections arguing on the novelty of the methodology.
- The trial NCT04680637 has initiated, the authors should update its status.

Is the work clearly and accurately presented and does it cite the current literature?

Partly

If applicable, is the statistical analysis and its interpretation appropriate?

Yes

Are all the source data underlying the results available to ensure full reproducibility?

Yes

Is the study design appropriate and is the work technically sound?

Partly

Are the conclusions drawn adequately supported by the results?

Yes

Are sufficient details of methods and analysis provided to allow replication by others?

Yes

Reviewer Expertise:

CD4 T cells, cytotoxicity, cancer, Innate lymphoid cells

We confirm that we have read this submission and believe that we have an appropriate level of expertise to confirm that it is of an acceptable scientific standard, however we have significant reservations, as outlined above.

# Response

**Published:** 2022-05-04

**Authors:** Ricardo Ferreira

**Affiliations:** University of Oxford, UK

We appreciate the Reviewer’s feedback and suggestions. We detail below the replies to the specific concerns raised by the reviewer:

  

- 1/ The main weakness of the study is the very limited number of subjects, which dampens the enthusiasm of the reviewer. Conclusions are drawn on 1 donor per study population (1 patient IFNhi, 1 patient IFNlo, 1 HD, with only limited clinical characteristics provided). It would have been highly recommended to validate the findings obtained by the transcriptomic/Proteomic analyses in additional samples from IFNhi and IFNlo SLE patients, by a flow cytometry based methodology.

*We agree that the main limitation of this study is indeed the very small sample size. Unfortunately, we do not have access to further clinical samples with ancillary clinical data about disease activity that can be used to validate these findings independently using either our single cell platform or a flow cytometry based methodology. We have included in the manuscript that some of our findings are consistent with recent findings in Sjogren’s syndrome, a related systemic autoimmune disease associated with a type I IFN response, where the authors have identified a similar CD4+ CTL population through scRNA-seq, and validated by flow cytometry by identifying a fraction of GZMB+ CD4+ T cells (see ref 20 – Hong X et al; Front Immunol 2021; doi: 10.3389/fimmu.2020.594658). Furthermore, we also cite two other studies where the authors have identified a similar subset of cytotoxic CD4+ T cells in blood by scRNA-seq in the context of bladder cancer (ref 19 – Oh DY et al; Cell 2020; doi: 10.1016/j.cell.2020.05.017) and in a cohort of supercentenarians (ref 21 – Hashimoto et al; PNAS 2019; doi: 10.1073/pnas.1907883116) and further validated the results using orthogonal methods, including flow cytometry. Given these results and the known reproducibility between FACS- and AbSeq-based protein quantification methods (see Mair F et al; Cell Rep 2020; doi: 10.1016/j.celrep.2020.03.063), we are very confident of the reproducibility of this phenotype and the presence of the cell subset in blood.*

*We note that the main motivation of this study was to provide a proof-of-principle for the use of a targeted scRNA-seq approach to better characterise the heterogeneity of the T and NK cell compartments. We opted to investigate these immune compartments in the context of SLE given our previous experience and data showing substantial alterations in the peripheral immune system of SLE patients with active disease. As stated above in the reply to Reviewer 1 (point #1), the key outstanding question is whether these immune alterations are maintained during the course of the disease, and particularly how they are regulated by increased IFN production during active disease. This study demonstrates that this approach could be well-suited to investigate these questions in a well-characterised longitudinal cohort of SLE patients. Moreover, it provides insight into specific T and NK cell subsets that could be altered in patients with active disease and that should be further monitored in SLE.*

*Nevertheless, we note that despite the sample size limitation, the increased resolution provided by this single-cell immunophenotyping approach, combined with the pre-selection strategy, provided a large number of T and NK cells profiled in this study. Furthermore, all samples were processed in parallel using sample barcodes, thereby eliminating most causes of technical variation that usually affect scRNA-seq experiments. These factors increase our confidence in the results reported in this study.*

*We state these limitations in the discussion and have now reduced the discussion section to provide increased focus on these limitations. We also state more clearly that this is a proof-of-principle study aiming at providing a resource for researchers to investigate the T and NK cell heterogeneity in SLE at the single-cell level.*

  

- 2/ Moreover, while the authors repeatedly stress the fact that they are utilizing a novel method combining protein and mRNA quantification, this type of analysis has also been largely used by others, eg., CITE-seq, on unsorted single immune cells, with good resolution and identification of discrete, rare immune cell states (such as CD4 CTLs).

*As the reviewer correctly points out, parallel assessment of mRNA and protein at the single-cell level is not novel. However, at the time of publication of our study these methods have been limited to whole-transcriptome scRNA-seq (ie. CITE-seq) on unsorted immune cells. The main novelty of this method is the combination of a cell subset pre-enrichment approach (by sorting of specific immune cell populations), combined with the design of a custom targeted mRNA panel. This approach substantially differs from the standard whole-transcriptome approaches. In this study we wanted to highlight these differences and strengths of the targeted approach. Importantly, whole-transcriptome methods tend to have very large sequencing requirements for the mRNA libraries, compared to our targeted method. Previously, we have shown that this targeted approach results in increased power to quantify the mRNA targets present in our custom panel (see Genome Med. 2020 Jun 24;12(1):55. doi: 10.1186/s13073-020-00756-z.), leading to improved clustering resolution. This has important implications on the use of the protein quantification on both methods: for traditional whole-transcriptome scRNA-sequencing, protein quantification (ie. CITE-seq) is therefore often restricted to a small panel of lineage-discriminating markers that are mostly used for cluster annotation purposes. In contrast, the reduced sequencing requirements in this targeted approach means that we can devote a much larger proportion of the budget to sequence the protein libraries, resulting in the use of much larger protein panels (including mostly proteins associated with cell function), which can be used to identify more subtle functional changes associated with protein expression changes.*

*Therefore, the two methods are complementary in their approach. We believe that the targeted approach could be well-suited for a wide range of researchers, as a hypothesis-based approach to immunophenotype larger numbers of cells from specific cell populations in a more cost-effective manner. The custom nature of the mRNA and protein panels makes this a more flexible approach, although it does have the inherent limitation of the target marker selection, which does not replace the discovery-based nature of the traditional whole-transcriptome scRNA-seq methods.*

*We agree with the reviewers that given the points above we should refrain from claiming novelty, and instead have edited the discussion to focus more on the strengths of the method and differences with more standard combined mRNA/protein quantification approaches at the single-cell level.*

  

- 3/ Finally, while the inclusion of a "stimulated" condition appears very interesting to the reviewer, the rationale to use PMA/Ionomycin instead of aCD3/CD28 activation (at least for T cell subsets) is unclear. This would have provided a more physiological activation, that would better reflect the in vivo events occurring in patients.

*The use of the PMA+Ionomycin activation was driven by previous experience in the lab, showing that very short stimulation (only 30-45 min instead of the 4-5h usually used for cytokine quantification at the protein level) induces a very distinct polarisation of the functional T _H_ lineages. This is mostly driven by the robust transcription of cytokine genes (as well as other activation-induced genes), which can be used to better dissect the heterogeneity of the sorted immune cell populations compared to the ex vivo (unstimulated) condition. Furthermore, as the reviewer points, since one of the motivations of this study was the characterisation of NK cells in SLE – particularly the rarer NK56br subset – we felt that a T-cell specific activation would not be best suited.*

*In our experience, activation with aCD3/CD28 provides a less robust induction of cytokine genes and requires much longer stimulations times as compared to PMA+Ionomycin. Nevertheless, we agree that for other studies, an aCD3/CD28 activation would be more suited and would yield very interesting data. We believe that this targeted scRNA-seq method is particularly well-suited to investigate more subtle changes associated with different immune perturbations and could therefore be adapted to a number of different experimental models and immune cell populations of interest.*

  

- 4/ By considering NK cells as CD56+CD127+ CD3- cells, the authors are including helper ILC3s in their sorting. This might represent a confounding parameter for the results' interpretation. Similarly, CD25-CD127+ CD4 T cells will also include Naive CD4 T cells, while in Fig 1B these cells are named Teff.

*One of the main advantages of single-cell methods over standard bulk assays, is that they are much more tolerant of cell heterogeneity. Indeed, single-cell methods will be able to identify small proportions of distinct non-expected “contaminant” cell populations, as long as they are sufficiently represented in the cell mixture. In contrast, in bulk cell assays, the output of the experiment may well be affected by varying degrees of contamination with different cell types.*

*Because of this, in this study, we deliberately delineated broader gates to define our target cell populations, to avoid potentially losing any specific immune cell population. In the specific case of ILC3 cells, we did not observe any specific cluster of cells sorted from the CD56+CD127+ CD3- gate (designated NK56br gate in this study), with a profile matching ILC3 cells. This does not exclude the possibility that a small number of ILC3 cells may have been sorted in that gate, but it does ensure that the numbers are negligeable and would not affect the interpretation of our results.*

*Regarding the CD25-CD127+ CD4+ T cell gate, we agree that the designation of “CD4+ CD127hi Teff” cells is confusing.  As the reviewer rightly points out, there is definitely a substantial proportion of naïve cells sorted from this gate (as annotated on the respective UMAP plots). We have therefore edited the text and figure to now refer to this population as “CD127hi Tconv” cells to more clearly differentiate from the converse CD127lowCD25hi Treg gate. Similarly, we changed the designation of the cells sorted from the “CD127low Teffs” to “CD127low Tconv” for consistency (as this gate was also shown to contain a small fraction of naïve CD4+ T cells).*

  

- 5/ Though nicely represented, the readability of several Figure panels is difficult (numbering of clusters Fig 2A, gene names and scale bar Fig 2B, blurry volcano plot Fig 3B,...).

*We have provided higher resolution files for our figures to increase the readability of the panels. We have also increased the font of the cluster names to improve legibility.*

  

- 6/ The discussion is very long, and highly speculative based on the findings obtained in the small patients cohort. I suggest refinement, specifically the sections arguing on the novelty of the methodology.

*As detailed above, we have now edited the discussion to shorten it and to refrain from claiming novelty of the methodology, but rather to more clearly detail the differences.*

  

- 7/ The trial NCT04680637 has initiated, the authors should update its status.

*We have edited the text in the revised version to reflect the change of status in the NCT04680637 trial.*

# Peer review: Reviewer response for version 1

**DOI:** 10.21956/wellcomeopenres.18624.r47452

**Published:** 2021-12-20

**Authors:** Nancy J. Olsen

**Affiliations:** 1 Division of Rheumatology, Department of Medicine, Penn State MS Hershey Medical Center, Hershey, PA, USA

This is an interesting and detailed study of the immune cell features in patients with systemic lupus erythematosus (SLE). The authors have shown the strength of combined transcriptomic and proteomic profiling in single-cell analyses. The findings suggest that standard lymphocyte subsets, such as CD4 and NK cells, are in fact very complex and that subsets of these cells, even rare ones, have functional features that likely contribute to disease pathogenesis. Strengths of this work include the detailed analyses, the very clearly presented data in multiple figures, and the fact that the proteomic results reinforce functional implications of the various phenotypes defined by flow cytometry.  

 The major weakness, which is discussed by the authors, is that this is a very limited analysis in terms of the patients sampled: one with high IFN signature, one with low IFN signature, and one healthy subject. The other available data on the two patients, levels of IL2 and circulating sCD25 are consistent with active disease. However, the IFN signature genes were not elevated as expected in the IFNhi individual. The explanation offered is that the IFN levels were determined at a separate time, prior to the sample taken for this study. This might imply that the cellular changes are more prolonged than the signature expression, but in the absence of longitudinal data, that is speculative. In addition, clinical features of the patients to confirm active disease are not described. The results will require further validation in larger patient samples, but the data presented suggest that this will be of interest for future studies. One additional note is that the NCT04680637 trial mentioned has now been started and is in recruiting phase; so that change might be made.

Is the work clearly and accurately presented and does it cite the current literature?

Yes

If applicable, is the statistical analysis and its interpretation appropriate?

Yes

Are all the source data underlying the results available to ensure full reproducibility?

Yes

Is the study design appropriate and is the work technically sound?

Yes

Are the conclusions drawn adequately supported by the results?

Yes

Are sufficient details of methods and analysis provided to allow replication by others?

Yes

Reviewer Expertise:

Autoimmune disease, systemic lupus erythematosus

I confirm that I have read this submission and believe that I have an appropriate level of expertise to confirm that it is of an acceptable scientific standard.

# Response

**Published:** 2022-01-05

**Authors:** Ricardo Ferreira

**Affiliations:** University of Oxford, UK

We appreciate the reviewer's feedback. We agree that the more relevant follow up question pertains to the duration of the IFN signature and how this tracks with the cellular alterations that we observe in this study. This can only be addressed in a longitudinal cohort, and hopefully this study will provide the rationale for monitoring CD4+ CTLs in future studies.

 We also note that although we do not observe a transcriptional IFN signature in the IFNhi patient, this is only true for the assessed T and NK cell subsets we profilled in this study.  We cannot comment on other myeloid populations, where the IFN signature is usually more pronounced and more likely to be detected for a longer period of time.

 We also appreciate the reminder about the status of the NCT04680637 trial, and will certainly edit the manuscript accordingly in the next version.

# Response

**Published:** 2022-05-04

**Authors:** Ricardo Ferreira

**Affiliations:** University of Oxford, UK

Update (4 May 2022):

*We have now edited the text in the revised version to mention that the NCT04680637 trial has started and initiated recruitment.*
